# Historical and Contemporary Perspectives on the Microbiological Aspects of Endodontics

**DOI:** 10.3390/dj6040049

**Published:** 2018-09-22

**Authors:** James L. Gutmann, Vivian Manjarrés

**Affiliations:** 1 Department of Endodontics, Texas A&M University College of Dentistry, Dallas, TX 75246, USA; 2 Department of Endodontics, Nova Southeastern University, College of Dental Medicine, Davie, FL 33314, USA; manjarre@nova.edu

**Keywords:** anachoresis, bacteria, biofilms, culturing, extraradicular infections, focal infection, history, microbiology, molecular biology

## Abstract

The microbiota of the oral cavity plays a significant role in pulpal and periapical diseases. Historically, 100 years ago little was known on microbiota, but after a century of investigations, only now can many of the intimate secrets of microbial growth, expansion, persistence, communal activities, and virulence be revealed. However, with the capabilities of the microbiota for mutation, quorum sensing, and information transference, researchers are hard-pressed to keep up with both the changes and challenges that an amazingly wide range of bacterial species pose for both the scientist and clinician. Fortunately, the development and expansion of a vast array of molecular biological investigative techniques have enabled dentistry and its associated medical fields to attempt to keep pace with the wide and fascinating world of oral microbiology.

## 1. Historical Perspectives

The colonization of micro-organisms/bacteria in the dental pulp via the carious process and their movement into the periapical tissues has long been known as the main etiologic factor in pulpal inflammation, necrosis, periapical periodontitis, and ultimately abscess formation. While micro-organisms had been identified by Antoni van Leeuwenhoek (1632–1723) in bacterial plaque, it would be close to 200 years before bacterial species would be related to both pulp disease and their potential spread to the entire body.

The world at large was alerted to the issue of the human mouth as a focus of bacterial infection, not only within the oral cavity, but also systemically by Willoughby D. Miller in 1888 and again in 1891 [1,2]. Miller was a graduate of the University of Pennsylvania Dental School and a disciple of Robert Koch (1843–1910) (a German physician and the founder of modern bacteriology and microbiology), having studied microbiology in Koch’s laboratory in Berlin. Miller’s purpose was to “call attention to the various diseases, both local and general, which have been found to result from the action of micro-organisms which have collected in the mouth, and to the various channels through which these micro-organisms or their waste products may obtain entrance to parts of the body adjacent to or remote from the mouth” [2]. Prior to that, numerous articles had been published in the German literature, with a smattering found in the United Kingdom, France, and the USA. Miller cultured and characterized bacteria from necrotic pulp and studied their pathogenic potential in animal experiments.

Subsequently, in 1900, in the British Medical Journal, Dr. William Hunter commented on bacteria and their presence in the oral cavity thusly; “the whole subject of oral sepsis as a cause of disease has been one of special interest to me for many years; that I have dealt with it at some length in published papers during the past year and a-half; and that the more I study it the more impressed. I am, at once with its importance, and with the extraordinary neglect with which it is treated alike by physicians and surgeons” [3]. Furthermore, to enhance his medical position on this topic, Hunter published a text entitled *Oral Sepsis* in 1901 [4] and a lengthy discussion on oral sepsis as a cause of disease in relation to general medicine in 1904 [5].

While these previous comments and publications set in motion issues surrounding the impact of oral sepsis for the dental profession, it was Dr. Hunter’s message that he delivered in a speech at the Faculty of Medicine at McGill University in Montreal on 3 October 1910 and which was later published in both Lancet [6] and Dental Cosmos [7] that was the springboard for the concept of “focal infection”, a term ultimately coined by Dr. Frank Billings and described as “a circumscribed area of tissue infected with pathogenic organisms” [8,9]. At this time the presence or toxic action of pyogenic organisms, such as staphylococci and streptococci in teeth via caries or the presence of extensive periodontal disease, whether symptomatic or not, served as the major battle cry to force eradication via tooth extraction.

At the Panama Pacific Dental Congress in 1915, Dr. JP. Buckley [10] echoed the words of HL. Ulrich saying that “[…] every tip of a devitalized tooth whether the root canal has been properly or improperly filled becomes a *locus resistancii minorii*” [11]. This stance on focal infection and its impact on systemic disease continued to be fostered by Dr. Weston A. Price with the publication of his two-volume research exposé on *Dental Infections—Oral and Systemic* [12] and *Dental Infections and Degenerative Diseases* [13] published in 1923. Along with the preaching of Rosenow [14] and Billings [15], the publications of Price resulted in a frightening era of tooth extraction for both the treatment of systemic disease and as a prophylactic measure against future disease states. The war against oral bacteria and their presence in the tooth or surrounding periradicular tissues was in full swing.

However, an evaluation of Price’s research determined it to be invalid in so many respects, as it did not include proper control populations but did include the use of excessive, uncontrolled doses of bacteria to prove his hypotheses. Price’s research was ultimately discredited over the next 20–30 years, and in 1951 the American Dental Association (ADA), through its official vehicle, its journal, took an extraordinary step in publishing a special edition reviewing the scientific literature and shifting the standard of dental practice to one of tooth retention, especially in the presence of a tooth with a non-vital pulp whenever possible [16].

An additional theory that emerged from the conflagrations of focal infection was the “hollow tube” theory. In their classic paper of 1931, Rickert and Dixon demonstrated “halos of irritation” around the open ends of implanted platinum and steel hypodermic needles [17]. For them this finding “gave rather convincing evidence that the circulatory elements diffusing out of the openings of these tubes were not well tolerated by the vital tissue” [17], and this was deemed as analogous to what occurred in pulpless teeth. Therefore, root canal systems required a hermetic seal to prevent the irritation and inflammation of the periapical tissues and possible spread of disease. In addition, the material used to fill the root canal or apical foramen could not irritate the tissues, and caution was expressed as to the use of many materials in root surgery, especially copper amalgam that was used in root-end fillings. While the need to prevent localized tissue irritation was valid, the hollow tube theory, as promulgated by Rickert and Dixon, was ultimately and effectively disproven by Goldman and Pearson [18], Torneck [19,20], and Phillips [21] in the 1960s.

Essentially, during the middle of the 20th century there was a call to arms to develop microbiologic assays to determine the exact nature of the bacteria that impacted on the dental pulp and periapical tissues, along with an enhancement of clinical techniques to identify and eradicate their presence. With the founding of the of the Philadelphia Root Canal Study Club in 1939 [22] in an era that was seeing the wholesale extraction of teeth driven by bacteria and based on the theory of focal infection, this one timely and fortuitous occurrence was the genesis for a national association of dentists interested in root canal therapy. Along with several other influential dentists, Louis I. Grossman began organizing the American Root Therapy Association in 1943. At the Chicago Dental Society meeting that year, 19 dentists from across the country met and the American Association of Endodontists (AAE) was officially formed.

During this period, authors, academicians and clinicians focused more on the issue of the demise of the dental pulp, with the presence of necrotic tissue and stagnant tissue fluids in the root canal as being the main cause of apical periodontitis. Many referred to this process as the strangulation of the dental pulp due to inflammation, swelling inside the pulpal space, and a shutting down of the vascular system, resulting in pulpal degeneration [23]. This theory could be traced back to 1894 when a dentist, Dr. AJ. Brown, presented a clinical case to support his premise [24]. This empiricism went uninvestigated until 1922; Dr. CJ. Grieves described the process of strangulation and pulpal demise as follows [25]:

“Any serious pulp disease generally induces intracellular pressure in the pulp chamber and thrombi or strangulation in the apical vessels; the pulp tissue degenerates […]” [25].

This concept appeared to be untenable by Boling and Robinson in 1938 [26], and ultimately this process was proven scientifically to be erroneous by Dr. Henry J. Van Hassel in his description of the circumferential spread of inflammation in the dental pulp and necrosis from a specific site of injury [27].

However, a new era in the role of micro-organisms, pulpal inflammation, and its spread into the periapical tissues was about to rise from the ashes of previous erroneous thought. Furthermore, the comment by Dr. Louis Grossman in 1940 indicating that it was disconcerting but true that practically every investigation dealing with the pulpless tooth (bacteria and their impact with the root canal) made prior to 1936 was invalid [28], was about to be realized. It was also at this time that there was focus on implementing a bacterial culture, for which most investigators floundered in their attempts to isolate both aerobic and anaerobic species (to be discussed in depth below). Listed in Table 1 is a partial but important listing of studies and investigators that in the early part of the 20th century sought to identify specific bacteria related to necrotic pulps and subsequent infection of the periradicular tissues [29,30,31,32,33,34,35,36,37,38,39,40,41,42,43,44,45,46,47,48,49]. 

Needless to say, their efforts often lacked specificity and the ability to culture the wide range of infecting organisms. Furthermore, when species were isolated it was often left the investigator to speculate about their activity in the root canal system. Moreover, relationships with other bacteria present in the root canal that were uncultivable due to shortcomings in technique or lack of proper culture media that might have favored specific species could not be determined (Table 2). Therefore, while some bacteria could be isolated, their relationship with pulpal demise and subsequent impact on the root-surrounding-tissues were still unknown.

Within the medical microbiological community in the 1950s, there was a concerted and extensive effort to identify bacteria using a specific antibody conjugated with fluorescein isothiocyanate [50]. This could be accomplished with simple bacterial smears. The smear technique was promoted by Prinz in 1928, but only to the extent that it would determine if a prepared root canal could be filled [51]. See Table 3 for Prinz’s smear technique.

However, it never seemed to become part of dental microbial identification pursuits, as it would not have been of benefit due to the unique environment of the infected root canal space and the shortcomings of bacterial sampling and limited laboratory identification techniques.

In 1965 a truly classical publication awakened the minds of the dental community and resulted in an explosion into microbiological research focused on dental caries, the dental pulp, and the spread of inflammation and infection to the supporting tissue. Kakehashi et al. [52] demonstrated the essential role of micro-organisms in the pathogenesis of periapical lesions in germ-free and conventional rats. Studies by both Torneck [20] and Makkes et al. [53] corroborated those of Kakehashi et al. [52], with further confirmation and enhanced data from the study of Möller et al. [54] using necrotic pulp tissue in the root canals of monkeys. The doorway to the full exploration of the microbiology of pulpal and periapical disease was opened wide for an exhaustive number of studies bolstered by sophisticated sampling and laboratory techniques. 

Winkler and van Amerongen [55] published in 1959 a rather extensive report on the bacteriologic results from 4000 root canals. At that time, “sterility” prior to root canal obturation was ideal, although rarely achievable or determinable, as clinical shortcomings in bacteriologic sampling were identified. Their results focused heavily on the presence of streptococci species, with all other organisms considered as chance contaminants. This only emphasized the variable issues encountered in using different culture media [35,38,56,57]. Quite possibly, the first extensive and meaningful microbiological examination of root canals and the periapical tissues of human teeth was published by Åke J. R. Möller in 1966 [54]. This ground-breaking study established the framework for future microbiological studies, in particular with his focus on obligate anaerobic species. He designed culture techniques to maximize the recovery of anaerobes and bacteria in small numbers under stress of root canal medicaments in treatment cases [58]. He also developed a transport medium (VMGA III) for these bacteria that would allow their growth until the sample could be processed in the laboratory. Unfortunately, the vast majority of endodontic post graduate and graduate students globally are totally unfamiliar with this work and how it impacted on the contemporary perspective of the focus of this paper. 

As mentioned previously, another issue that fits into the historical perspective is the bacterial culture of the root canal and the wide range of issues, confusions, and challenges that it posed. This will be discussed in transitional perspectives below.

## 2. Transitional Perspectives

TW. Onderdonk [59] may have been the first to suggest the possibility of culturing root canals to determine whether bacteria were present prior to filling “[…] I would suggest […] the insertion of a root-treatment on cotton or paper points, completely filling the canals, sealing the cavity with a temporary filling, completing the operation at the next visit if the tooth is comfortable […] I wish to suggest [also] what I am pleased to call the scientific test—i.e., having a culture made from the cotton dressing, also from a fresh specimen taken after a thorough disinfection when we think the root is ready to fill and, with the aid of the bacteriologist, know when we have secured an aseptic root” [59].

Almost 20 years later, Coolidge recommended that culturing should be adopted as a routine clinical procedure during root canal procedures [60]. The use of cultures to demonstrate the bacteriologic status of root canals prior to obturation overcame the criticism of root canal procedures during the focal infection era and contributed substantially to the recognition of endodontics as a specialty of dentistry [61]. 

The philosophy of culturing as an integral procedure developed further when there was much concern regarding the treatment of pulpless teeth, which may serve as a cause of systemic illness. However, if the focus of infection was present within the root canal and that focus was removed, a pulpless tooth could not cause focal infection, as described by Fish [62,63]. 

In the 1940s, Grossman developed two intracanal medicaments to disinfect the root canal system; penicillin, bacitracin, streptomycin, and sodium caprylate (PBSC) and penicillin, bacitracin, streptomycin, and nystatin (PBSN) [64]. These were in paste form and he found that during the culturing process, some of the medicament was being transferred into the culture medium, thereby eliminating the growth of the sampled organisms [28]. See Table 4 for Grossman’s culture technique.

Buchbinder and Bartels [65] in 1951, along with Bender and Seltzer [66] in 1954 also registered their concern regarding the occurrence of false negative cultures that resulted with the transference of the medicaments (primarily streptomycin and chloramphenicol) into the culture media, and cautioned that the bacterial culture method of evaluation should be held in abeyance until some agent appears that promises to neutralize the effect of the medicaments in a medium. In particular, bacitracin was identified by Grossman as the main culprit in this process [67] and he recommended an alteration in the sampling technique that consisted of a least the use of three paper points to remove the paste prior to the final sampling of the root canal. 

Crawford and Shankle undertook an extensive study applying newer methods to study the microbiota of the infected root canal in 1961 [68]. They examined both open teeth and closed teeth and used microscopic methods, agar plating and thioglycolate medium. Phase contrast, dark-field, and bright-field microscopy were used, along with aerobic and anaerobic cultures on agar. They reaffirmed that microorganisms could be detected microscopically that could not be detected by culture alone.

Many investigators in the early to middle of the 20th century considered bacteriologic sampling an important and necessary aspect of root canal procedures.

Although it had become an accepted principle that one must obtain prefilling “sterility” of root canals, as demonstrated by the culture method, in order to achieve success following root canal obturation, the concept and value of culturing during root canal procedures was criticized by Seltzer et al. [69]. The results of their clinical studies indicated that there was no difference between the success rate in teeth with a positive prefilling culture and that in teeth with a negative prefilling culture [70]. Two philosophical and clinical camps arose in the endodontic sector; one that based their argument on achieving higher clinical success rates in teeth with negative prefilling cultures, and another that claimed clinical studies showed no difference in the prognosis of the cases with or without a negative culture, and that the technique as practiced was inadequate to identify the true microbial flora of the root canal. This led to a rethinking of the culture technique with a greater focus on both the organisms implicated and the techniques required to identify them.

In 1970 Morse presented a critical evaluation of the culturing technique. His stated support for this concept can be found below in the bulleted points [71].

- A culture is a dependable indicator of the microbiological status of the root canal
- Use of the culture acts as a check on an aseptic technique
- Culturing is useful for teaching purposes
- The use of the culture technique impresses the patient as being “scientific”
- The culture provides a check on the thoroughness of the canal preparation
- A negative culture allows for filling at the next visit

Within this framework of attributes to support culture there was, however, a plethora of limitations. Refutations for this concept were as follows [72]:

- Sampling for bacteria was significantly hindered by inaccessible areas of the root canal system
- Some critical species could be lost in transfers, allowing for overgrowth of opportunistic bacteria and contamination of the sample
- Lack of adequacy and appropriateness of the culture media, and failure to provide the necessary growth factors to enable bacteria to survive once they were obtained from the root canal
- Species could be uncultivable based on the techniques and materials used
- Bacterial identification was expensive, time-consuming, and for many species their taxonomy could not be defined

The biggest transitional perspective in this controversy focused on: (1) the identification and characterization of the entire spectrum of bacterial species found in the infected root canal; (2) how they obtain access to the root canal; (3) where they are positioned in the root canal; (4) what makes them become invasive and virulent; (5) how they survive in the root canal; and (6) how they exit and impact on the periradicular tissues, even after what would be considered as a quality root canal procedure. Clinical culturing techniques required sophistication and specific culture media to identified the species identified. No one culture medium could provide the identification of such a wide range of species [73,74]. Often culturing techniques came under fire and controversies surrounded the value of the culturing, with many clinicians abandoning these procedures. Due to the conflicting nature of published research and the adamant position of many academicians and clinicians, key studies that sought to clarify the bacteriologic status of the infected root canal rose to prominence. 

In early 1970s, Berg and Nord proposed the use of a method for the isolation of anaerobic bacteria from root canal specimens, using continuous anaerobiosis [75]. The focus was to ensure that no specimens would be lost in the culturing or transfer techniques. A mobile anaerobe unit that supplied a gentle stream of oxygen-free gas over the tooth was used when the bacteriologic sample is taken. Paper-points were then, under oxygen-free gas, immediately transferred to pre-reduced, anaerobically sterilized roll tubes. The different isolated anaerobic bacteria were then typed with biochemical tests, antibiotic susceptibility testing, and gas chromatographic analysis of volatile fatty acids and alcohols. A highly successful recovery of anaerobic bacteria was realized with this technique.

In the mid-1970s, Zielke and coworkers [76] established further the importance of anaerobic culturing, while Sundqvist [77], in his break-through research on the identification of bacterial species in intact teeth with lesions, identified a vast array of species using anaerobic culturing techniques during sampling, transportation and cultivation. With the supportive data provided by Wittgow and Sabiston [78] and Kantz and Henry [79] in the mid-1970s, the uniqueness of culturing techniques in sampling, transference and cultivation in supportive media was emphasized by Sundqvist in 1976 [77], where he showed the domination of anaerobic bacteria in intact teeth with necrotic pulps. The approach to culturing had taken a significant leap forward, as had the rapid identification of heretofore unidentified bacterial species. This also opened the flood gates to the understanding of interactions of bacteria in the root canal system and broadened the scope in our understanding of both virulence factors and host responses. 

## 3. Contemporary Perspectives

Somewhere between 300 and 700 bacterial species can be found in the oral cavity, with any particular individual harboring 100–200 of these species (Table 5) [80,81,82,83,84,85,86,87,88,89,90,91].

The numbers are dependent on the nature of the study and the techniques used to identify the microbiota. However, only a limited number have been consistently isolated from root canal infections [82,92]. The uniqueness of this occurrence may lie in the fact that within the functional groups of organisms whose metabolism is compatible with the root canal, there is no host restriction via the immune system, because the pulp is dead and the space is inaccessible to inflammatory cells. Thus, although only a quarter of oral bacterial species can colonize the root canal and cause infection, it appears that a variety of permutations of species within that group may be present [82].

Once the root canal is infected coronally, infection progresses apically until bacterial products or bacteria themselves are in a position to stimulate the periapical tissues, thereby leading to apical periodontitis. The apical part of the root canal system drives the selection of a more diverse and more anaerobic community than the coronal part [93]. The presence of a distinct ecological niche in the apical region explains the difficulty of eradication of the infection and the impact that it has on the development of periapical periodontitis. By the very nature of the apical environment, and the high bacterial diversity in this region [94], the persistence and presence of biofilms are conceivably the most important aspect of this development beyond the confines of the root canal [95,96]. 

Endodontic, or more appropriately, specific pulpal and periapical infections, reflect a polymicrobial nature, with obligate anaerobic bacteria conspicuously dominating the microbiota in primary infections. There are various microorganisms related to intraradicular and extraradicular infections (see below Section 3.4) and organisms involved in persistent infections, and more likely than not they are protected by biofilms. 

Due to the vast number of microbial studies from the early 1960s forward, it became painfully obvious that at least 50% of the oral microbiota were uncultivable [97,98]. By a priori reasoning it would seem that the same could be said of the microbiota of the infected root canal system. However, within the root canal system the ability of many of the species to survive may be highly dependent on environmental needs, the presence of supportive bacteria and biofilms. Furthermore, many species may be in a dormant stage or not be cultivable using routine techniques for anaerobes (viable but not culturable, VBNC). This initially created many challenges to the investigations of infected root canal microbiota and led ultimately to the use of molecular biological techniques. 

One of the first techniques that revolutionized microbial identification was the PCR or polymerase chain reaction technique [99]. The use of this technique has expanded greatly into many different forms and along with a multitude of other molecular biology techniques has enabled the in-depth identification and characterization of bacterial species found in the root canal system (Table 5). The use of these laboratory strategies (Table 6) enables the determination of VBNC microorganisms, including the delineation of bacteria species located in biofilms [100,101,102]. 

The majority of molecular tests in use today are qualitative tests. Qualitative tests are best suited for the detection of microorganisms in specimens whose presence, at any level, is associated with a disease state, which is appropriate for the challenges encountered in the root canal and beyond. This includes microorganism that are not regarded as normal flora, as well as any organism isolated from a sterile site. Interestingly, a wide range of bacterial species (microbiome) were identified in presumably healthy pulps using genomic DNA and 16s ribosomal RNA PCR primers and MiSeq sequencing [103]. While the model used in this study could be challenged, the findings do demonstrate the ubiquitous nature of bacteria in previously-thought-to-be hallowed ground, as bacterial genetic material was successfully isolated, amplified and sequenced from 100% of the pulp tissues sampled with an average of 343 unique taxa per sample. To fully understand and appreciate the wide range of molecular biological testing that is being used today, the reader is referred to the review by Buchan and Ledeboer [104].

While there are a large number and variety of molecular techniques available for the identification of microorganisms from the infected root canal, it would be remiss to dismiss the value of the traditional culture, as none of the molecular techniques are without flaws [97]. As indicated by Spratt [99], culture-dependent and culture-independent techniques are not exclusive to each other and should be used together to understand the complex nature of root canal infections. Therefore, despite technological advances in laboratory diagnostics, the clinical microbiology continues to rely heavily on traditional methods, including culture, phenotypic, and biochemical tests, to identify microorganisms present in clinical specimens. For specimens which are still best analyzed using culture, automation of primary processing and plating, coupled with initial culture examination aided by high-resolution optics, has reduced time spent on mundane tasks associated with the initial steps of clinical bacteriology and improved efficiency. Meanwhile, rapid and accurate identification of these cultured microorganisms is made possible using mass spectrometry (MS). 

Today’s range of identified bacterial species is a far cry from what it was 75–100 years ago, when streptococci, micrococci, lactobacilli, and staphylococci and their range of species were thought to be the sole inhabitants in root canal infections.

### 3.1. Anachoresis

“Anachoresis”, as defined by Robinson and Boling is that phenomenon by which blood-borne bacteria, dyes, pigments, metallic substances, foreign proteins and other materials are attracted to, and fixed in, circumscribed areas of inflammation [105]. While the concept of anachoresis was prevalent in the medical community in the early 1900s, its role in pulpal demise was first suggested by Csernyei from Milan, Italy in 1939 (who referred to it as “anacoric”) [106] and was reaffirmed by Robinson and Boling in 1941 [105]. This all came on the heels of the “focal infection” era, during which circumspect chronic periapical inflammations and dental granulomas had long been considered as foci for various systemic diseases. Csernyei discerned that chronic periapical inflammations have an anacoric effect on microorganisms and that organisms do not remain in the blood but take refuge in the area of inflammation [106]. 

“The root ends of teeth with necrotic and putrefied pulps are surrounded by chronically inflamed and infected tissues which may receive bacteria hematogenously. Since the tissues remain chronically inflamed after antiseptic root canal therapy, by anacoresis they may become foci for reentry of bacteria and toxins into the blood stream even after immigration from root canal has ceased” [106].

In an animal (dog) study, Gier and Mitchell affirmed the concept on anachoresis, as they found that artificially injured pulps harbored hematogenous bacteria that had been injected into the animals prior to the injury [107]. The inflammatory reaction of the pulp was proportionate to the degree of tissue compromise in the experimental design. The greater the degree of injury, the more readily was infection demonstrated. Today, the concept of anachoresis has neither been proven or disproven beyond a doubt and should still be considered within the realm of bacterial infections in compromised tissues [108].

### 3.2. Biofilms

Author’s note: Due to the fact that research on biofilms has had a similar evolution in thought and basis in the literature and that its science is expanding rapidly, codified concepts will be presented and are supported by the literature cited at the end of this section. By the time this manuscript is published, the important issues surrounding biofilms and their impact on pulpal and periapical disease states may very well have assumed a totally different profile.

Biofilms are highly organized structures consisting of bacterial cells enclosed in a self-produced extracellular polymeric matrix attached on a surface. Biofilms may also be considered as a layer of condensation of microbiota or a microbial-derived community consisting of cells that are irreversibly attached to a substratum or interface or to each other, and embedded in a matrix of extracellular polysaccharides in addition to extracellular DNA (eDNA) and extracellular protein. To accomplish this physical state, microorganisms must achieve four key biological characteristics;

1. Self-organization (autopoiesis),
2. Resistance to environmental perturbations (homeostasis),
3. Exhibition of synergy, and
4. Responsiveness to environmental changes as a unit in communal fashion.

Bacteria can form biofilms on any surface that is flooded with a nutrient-containing fluid consisting of bacterial cells, a solid surface, and a fluid medium. The established mechanisms for biofilms to form occur in three additive stages. Firstly, adsorption of inorganic and organic molecules to the solid surface occurs, leading to the formation of conditioning layer. Secondly, there is adhesion of microbial cells to the conditioned layer, with attachment affected by pH, temperature, surface energy of the substrate, nutritional availability, time of contact of bacteria, bacterial cell surface charge, and surface hydrophobicity. The bacterial substrate interaction occurs sequentially in three phases:

- Transport of microbe to substrate surface which is mediated by fimbriae, pili, flagella and extracellular polysaccharides (glycocalyx).
- Initial non-specific microbial–substrate adherence which occurs due to combination of electrostatic attraction, covalent and hydrogen bonding, dipole and hydrophobic interaction.
- Specific microbial substrate adherence phase. In this phase, adhesin or ligand on the bacterial cell surface binds to receptors on the substrate.

Thirdly, development and expansion of the biofilm will inevitably occur provided there is no active disruption in its life cycle. In this stage, a monolayer of microbes may attract secondary colonizers forming a microcolony, and the collection of microcolonies gives rise to the final structure of biofilm.

Bacteria that are part of a biofilm have some unique advantages as they are able to survive in stringent and demanding environmental conditions, an existence that is offered primarily by the biofilm’s extracellular matrix. This is due to the organized internal compartmentalization in the biofilm that allows bacteria possessing different growth requirements to survive in their own microenvironments. Benefits include protection from environmental threats, enhanced tolerance to antimicrobials, and the ability of the bacteria to communicate cell-to-cell (quorum sensing), although verification of the nature and mechanisms of this activity in the root canal is lacking in research. 

The establishment and persistence of biofilms in the root canal system is the primary source for the development of chronic periapical periodontitis and most likely highly resistant extraradicular infections. The latter can occur when biofilms expand and form around the apical foramen, attached to the cementum. Furthermore, as biofilms develop in different areas of the root canal system, they serve as the initiators for the movement of bacteria into the dentinal tubules, depending on tubule patency [109,110,111,112,113,114,115,116,117,118,119,120,121,122,123,124,125,126,127,128,129,130,131,132].

### 3.3. Dentinal Tubules and Bacteria

Once bacteria have invaded the dental pulp and root canal space, they may also invade the dentinal tubules [118,122,133]. This is particularly important for bacterial survival in the radicular portion of the canal, where the anatomy can be highly complex [134,135] and favor bacterial penetration, adhesion, and isolated biofilm formation [136]. During this process of penetration, bacterial species may very well compete for entry into the tubules and specific bacteria may assist others in doing so [118,137], while other specific peptides derived from collagen may be inhibitory to the penetration of other species [138]. However, the apical environment heavily favors the growth and survival of the obligate anaerobic species deep in the dental tubules [133,139,140,141]. As such, the presence of these species have been implicated in recurrent periapical disease and treatment failure [142]. The clinical implications of this set of circumstances creates major challenges in the disinfection and eradication of both the bacteria and their established biofilms in the apical region. In itself, the positioning of bacteria in the tubules may pose unique challenges anywhere along the length of the root canal and may serve as reservoirs of infection in those cases where the quality of the root canal procedures is suspect or where there may be concomitant periodontal disease.

### 3.4. Extraradicular Infections

While the characterization of the intracanal infecting organisms has received a great deal of attention, for example through perspectives on biofilms most recently, the expansion of the infectious process to the periapical/periradicular tissues has also been widely investigated with a number of investigative techniques. Historically it was thought that bacterial species, while present in the root canal, did not invade the periapical tissues unless specific virulence or invasive factors were present [143,144]. Tronstad et al. brought the concept of extraradicular infections and their salient ramifications to the endodontic community in the latter part of the 20th century [143,145]. However, extraradicular infections are now recognized as common occurrences in asymptomatic teeth with apical periodontitis, which has been verified with molecular biologic techniques [144]. Presently, with the understanding of biofilms and their development in the apical regions and expansion on to the apical cementum, essentially the presence of apical periodontitis is the extension of pulpal disease and root canal infections with vast array of organisms, many of which are well-known and fastidious, and many of which are yet to be identified. The predominant organisms present have been identified as obligate anaerobes [121,143,144,145,146,147,148,149,150,151], such as;

- *Fusobacterium nucleatum*
- *Actinomyces* spp.
- *Prevotella* spp.
- *Treponema* spp.
- *Porphyromonas endodontalis* and *gingivalis*
- *Propionibacterium propionicum*
- *Streptococcus*/*Staphylococcus*
- *Parvimonas micra*
- *Bacteroides ureolytiocus*
- *Clostriduim botulinum* and *sordelli*
- *Campylobacter*
- *Pseudoramibacter alactolyticus*

However, there is evidence that extraradicular infections may also be independent of the root canal and its microbial inhabitants [152]. Many investigators have focused on *Actinomyces* spp. and *Propionibacterum* as being major players because of their role in biofilm formation [149,153,154].

The most frequent bacterial species identified in different studies, using different techniques may vary considerably, while the presence of some species of microorganisms seems to be somewhat determinant. However, the true origin of extraradicular infection is still not fully characterized, because there are marked differences in methodologies, materials, aims, and techniques, which has led to variable and heterogeneous outcomes. Extraradicular infection is likely a multifactorial disease that requires further systematic investigation using standardized techniques [155].

Interesting periapical findings identified among a wide range of investigators using tools/tests such as histopathology, histobacteriology, scanning electron microscopy, stereomicroscopy, radiography, and PCR were the presence of mineralized biofilms, plaque, irregular areas of resorption-like craters or lacunae at different depths in which bacteria and biofilm persisted, specific granular entities and the presence of sinus tracts [156,157,158,159,160,161]. These findings would account for the persistence of apical periodontitis and create challenges in the clinical attempts to remove these irritants nonsurgically. In fact, under many circumstances the choice of nonsurgical management, given an understanding of apical anatomy and the persistence of both intraradicular and extraradicular biofilms, would be contraindicated in favor of a surgical approach, even in the presence of symptom-free lesions [145,162,163]. 

Actinomyces species identified in extraradicular infections may also have a special role in the development of the sulfur granules in periapical granulomas [144,153]. As in dental plaque, these organisms may serve as pioneer, advancing bacteria that build scaffolds so that other bacterial species are attracted and may establish themselves at the site. A biofilm then develops, in these instances in the form of granules in the tissue.

## 4. Observations and Conclusions

While significant advances have been made in the past 50 years in the scientific and clinical study of microbes, their identification, their impact on the dental pulp and periapical tissues, and their eradication, the endodontic community is still faced with a plethora of challenges. One of the biggest challenges is the root anatomy itself, as it can harbor bacterial species, especially within biofilms, allowing them to grow, expand and infect the periapical tissues, oftentimes without immediate patient signs or symptoms. Many of these species may go undetected due to sampling procedures or protection within the community of biofilms. For example, one of the bacterial species that has been implicated in the failure of root canal procedures is *Enterococcus faecalis* [164,165,166]. As such and because of its fastidious nature and adaptability in the root canal, it has been established in research circles as the “bug to kill” to determine the efficacious nature of the treatments being rendered. However, it has the propensity to colonize deeply in biofilms and be resistant to a wide range of antimicrobials and clinical procedures; hence the root canal anatomy also plays an important role in its persistence. Therefore, *E. faecalis* is frequently recovered from secondary persistent infections associated with treatment failures [167,168], and for that matter, cases that appear to be treated properly may also fail due the persistence of this organism. Ultimately it can result in invasion into the periradicular tissues, with subsequent development of abscesses and diffused infections (cellulitis) [169]. Clinically, despite aggressive shaping and cleaning during root canal procedures, these bacteria may persist in 20 to 33% of root canals [170]. The frustrating rates of posttreatment disease are mainly attributed to the limitations of the present technologies that offer no tools to combat intracanal *E. faecalis* biofilm infection [167,168]. However, a perplexing question still arises. What is the source of these species? They are not readily identified in primary infections of the root canal. According to Vidana et al. [171], infections with *E. faecalis* are probably not derived from the patient’s own normal microflora, which indicates that these infections are of exogenous origin for which the implications surrounding contamination from poor restoration, cracks in teeth, exposed dentinal tubules, and periodontal disease have to be considered. To combat this type of bacterial infection, knowing the challenges faced with standard techniques, a unique phage therapy has been proposed by Kalifa et al. [166].

Finally, to paraphrase Fouad [91], the frontiers of microbiological research in pulpal and periapical diseases are vast and have not been adequately addressed. These include the rapid molecular identification of antibiotic resistance to permit the effective use of antimicrobials; interactions that occur in acute and chronic infections given the array of identified and VBNC organisms; the body’s ability to heal to a certain extent even in the presence of bacteria; identification and management of the initial bacterial insult from caries to a full-blown abscess; and the full characterization of bacterial virulence factors and their subsequent interaction with host responses in both the healthy and medically challenged patient. Once these issues and others are resolved, the choices in therapeutic or technical management will be readily at the disposal of the clinician.

## Figures and Tables

**Table 1:** Partial listing of studies and investigators—early part of the 20th century and prior to 1960.

Author	Year	Reference
La Roche M.	**1918**	The importance of bacteriologic findings in relation to the treatment of infected teeth. *J. Allied. Dent. Sci. 13*, 154–156 [29].
Appleton Jr., JLT.	**1927**	Bacteriologic control of the treatment of periapical infection. *Dent. Item Int. 49*, 589–597 [30].
Bullreid A.	**1931**	Bacteriologic studies of apical infection. *Brit. Dent. J. 52*, 105–114; 145–151 [31].
MacPhee G.	**1936**	The problem of the pulpless tooth. *Brit. Dent. J. 60*, 119–125 [32].
Prader F.	**1937**	*Der bakteriologische Test in der Wurzelbehandlung*. *Schweiz Mschr Zahnheilk 47*, 35–59 [33].
Kanner O.	**1938**	How may bacteria enter the pulps of intact teeth. *J. Dent. Res. 17*, 47–52 [34].
Buchbinder M.	**1940**	Results of culturing root canals by simple anaerobic method. *J. Dent. Res. 19*, 426 [35].
Morse Jr., FW, Yates MF.	**1941**	Follow-up studies of root filled teeth in relation to bacteriologic findings. *J. Am. Dent. Assoc. 28*, 956–971 [36].
Hayes R.	**1943**	Clinical and bacteriological study of 340 pulp therapy cases. *J. Dent. Res. 22*, 301–307 [37].
Shay D.	**1947**	The selection of a suitable medium for culturing root canals. *J. Dent. Res. 26*, 327–333 [38].
Appleton Jr., JLT.	**1950**	Bacteriologic Infection with Special Reference to Dental Practice. Lea and Febiger, Philadelphia [39].
Alin K., Agren E.	**1954**	The bacterial flora of odontogentic infections and its sensitivity to antibiotics. *Acta Odont. Scand. 12*, 85–98 [40].
Cran JA.	**1954**	I. Study of the pathology and bacteriology of the pulpless tooth and its bearing on treatment. *Aust. J. Dent. 58*, 291–296 [41].
Cran JA.	**1955**	II. Study of the pathology and bacteriology of the pulpless tooth and its bearing on treatment. *Aust. J. Dent. 59*, 82–84 [42].
Cran JA.	**1956**	III. Study of the pathology and bacteriology of the pulpless tooth and its bearing on treatment *Aust. J. Dent. 1*, 161–164 [43].
Leavitt JM., Naidorf IJ., Shugaevesky P.	**1955**	Aerobes and anaerobes in endodontics. Part I. The undetected anaerobes in endodontics. Part II. Sensitive culture medium for the detection of both aerobes and anaerobes. *NY State Dent. J. 2*, 377–382 [44].
Strindberg LZ.	**1956**	The dependence of the results of pulp therapy on certain factors. An analytic study based on radiographic and clinical follow-up examination. *Acta Odonto. Scand. 14*, *Suppl 21*, 1–175 [45].
Engström B., Frostell G.	**1957**	A study of the bacteriology of the non-vital pulp in cases of intact pulp chambers. *Svensk. Tandläk. T. 50*, 287–301 [46].
Glasser MM.	**1958**	Bacteriologic control in endodontics. *Oral Surg. 11*, 1278–1283 [47].
Grossman LI.	**1959**	Bacteriologic status in periapical tissues in 150 cases of infected pulpless teeth. *J. Dent. Res. 38*, 101–104 [48].
Shovelton DS., Sidaway DA.	**1960**	Infection in root canals. *Brit. Dent. J. 108*, 115–118 [49].

**Table 2:** Historically identified culture media.

Brain–heart infusion broth (BHI)
Trypticase dextrose/soy broth
Brewer’s thioglycolate medium
Serum dextrose broth
Hormone broth
Rosenow’s glucose brain broth
Rosenow’s liver broth
Glucose ascites broth
Prereduced supplemental brain heat infusion (PRS)
Oxoid BHI medium
Robertson’s cooked meat medium
Tryptose starch yeast extract

Investigators would alter the media with specific growth factors.

**Table 3:** Details and techniques of Prinz’s microscopic bacterial smear technique** [51].

Smear widely and evenly over a clean slide the dressing obtained from a root canal immediately upon it removal. Dry smear in air. Fix by passing rapidly through flame three times. Stain for three to five minutes with Löffler’s alkaline methylene-blue solution. Wash off stain with tap water. Invert cover-glass on slide film side down. Blot dry. Place drop of cedar oil upon stained film. Examine with oil immersion lens (1.9 or 1/12 inch).

** Prinz, H. *Diseases of the Soft Structures of the Teeth and Their Treatment*, Philadelphia, 1928, Lea & Febiger [51].

**Table 4:** Details and techniques of Grossman’s culture technique** [28].

Discard dressing from the previous technique. Dry the canal with sterile absorbent points, and then take a culture with a fresh absorbent point. Inoculate the culture medium located in test tubes. Incubate in electric incubator or submerge the apical half of the culture tube in 98° water. Examine the tubes for turbidity (growth) or no growth (clear liquid).

** Grossman, L l. Root Canal Therapy. Lea & Febiger; Philadelphia*:* **1940**. [28].

**Table 5:** Composite of multiple studies that provide a wide range of root canal microbiota [80,81,82,83,84,85,86,87,88,89,90,91].

**Gram-negative anaerobic rods**
*Prevotella* *Porphyromonas*
**Gram-positive anaerobic rods**
*Pseudoramibacter alactolyticus* *Filifactor alocis* *Slackia exigua* *Actinomyces* spp. *Propionibacterium propionicum* *Eubacterium* spp. *Olsenella* spp. *Mogibacterium timidum*
**Gram-positive cocci**
*Parvimonas micra* (previously *Peptostreptococcus micros* or *Micromonas micros*) *Streptococcus* spp. *Streptococcus anginosus* *Streptococcus mitisi* *Streptococcus sanguinis* *Enterococcus faecalis* (primarily secondary infections)
**Spirochetes**
*Treponema* spp.
**Species present in variable amounts**
*Campylobacterspp* (Gram-negative anaerobic rods) *Campylobacter rectus* and *Campylobacter gracilis.* *Catonella morbi* (obligate anaerobic Gram-negative rods) *Veillonella parvula* *Capnocytophaga gingivalis* *Centipeda periodontii* *Eikenella corrodens* *Granulicatella adiacens* *Neisseria mucosa* *Gemella morbillorum* *Corynebacterium matruchotii* *Bifidobacterium dentium* and anaerobic lactobacilli *Tannerella forsythia* (previously *Bacteroides forsythus)* *Dialister* (asaccharolytic obligately anaerobic Gram-negative coccobacilli) *Fusobacterium nucleatum* and *periodonticum*
**Viable but not routinely culturable (VNBC)**
*Dialister* oral clone BSO16 *Migasphaera* oral clone BSO16 *Solobacterium* *Olsenella* *Eubacterium* *Cytophaga* Bacteroidetes oral clone XO 83 Lachnospiraceae oral clone 55A-34 Lachnospiraceae oral clone MCE 7–60 *Veillonella* oral clone BP 1–85 *Prevotella* oral clone PUS 9.180 *Eubacterium* oral clone *BP 1–89 and* Other microorganisms in pulpal/periapical infections *Synergistes* spp.
**Fungi**—**Viruses**
*Candida albicans* Herpes spp.

**Table 6:** Partial listing of molecular biological tests and techniques (some of these tests have multiple names as used by various investigators).

Polymerase chain reaction (PCR) Real-time PCR (RT-PCR) Quantitative PCR (qPCR) Multiplex PCR Species-specific PCR Nested PCR Microarray Checkerboard DNA (DNA hybridization) Stand displacement amplification Transcription-mediated amplification Helicase-dependent amplification Two-stage nested PCR Whole-genome sequencing (WGS) Matrix-assisted laser desorption ionization–time of flight (MALDI-TOF) MS Digital PCR Next-generation sequencing (NGS) Nucleic acid sequencing Nucleic acid amplification tests (NAAT) using thermostable polymerase (PCR) Loop-mediated isothermal amplification (LAMP) Helicase-dependent amplification (HDA) Transcription-mediated amplification (TMA) of a nucleic acid target Mass spectrometry (MS) Fluorescence spectroscopy

Note: Many of these tests have pushed the landscape of molecular diagnostics further, allowing for analysis of complex, polymicrobial specimens and enabling accurate quantification of organisms present as <0.01% of the microbial consortium in a specimen. Recent applications of these advanced techniques can be found in [100,101,102].
